# External focus strategy improves visuomotor control of gait in older adults

**DOI:** 10.1007/s00426-025-02122-3

**Published:** 2025-04-23

**Authors:** Toby C. T. Mak, Thomson W. L. Wong, Melody C. Y. Leung, Duo W. C. Wong, Debbie C. L. Chan, Shamay S. M. Ng

**Affiliations:** 1 Department of Rehabilitation Sciences, The Hong Kong Polytechnic University, Kowloon, Hong Kong S.A.R., China; 2 Department of Biomedical Engineering, The Hong Kong Polytechnic University, Kowloon, Hong Kong S.A.R., China

## Abstract

**Purpose:**

Few studies have adopted external focus strategies to mitigate the negative effects of conscious movement processing in older adults. We investigated whether a single-session intervention (SSI) using an external focus could improve gait stability and visual search behaviors during adaptive locomotion in older adults.

**Methods:**

We randomly allocated 112 older adults to either an external focus (EXT, *n* = 56) or a control group (CON, *n* = 56). Participants performed an obstacle circumvention walking task along an 8-m walkway for five trials at pre-intervention (T0), post-intervention (T1), and retention (T2). The training phase consisted of 20 walking trials with obstacle circumvention. EXT focused on digits displayed on monitors at their path destinations during walking, while CON walked naturally without any specific instructions. Gait kinematics (i.e., gait variabilities and body sway) and visual search data were collected at T0, T1, and T2.

**Results:**

Only EXT reduced body sway and variability of spatial and temporal gait parameters, while increasing gait speed when comparing T1 and T2 to T0. EXT also reduced the number of visual fixation and fixation duration percentage on the ground while increasing fixation duration percentage on the destination when comparing T1 and T2 to T0.

**Conclusions:**

This study is the first to explore SSI with an external focus in older adults, providing evidence of significant improvements in gait stability and visual search behaviors that facilitate feedforward planning. Practicing with an external focus strategy could be recommended as an adjunctive psychomotor approach in clinical settings to enhance visuomotor performance in older adults.

**Supplementary Information:**

The online version contains supplementary material available at 10.1007/s00426-025-02122-3.

## Introduction

Maintaining a steady gait is critical for older adults to remain independent and undertake everyday activities with a lower risk of falling (Hamacher et al., 2011). From a psychomotor perspective, researchers discovered that older adults often tend to consciously monitor and control their walking movements when they are motivated to walk safely (e.g., to avoid falling) (Masters et al., 2007; Orrell et al., 2009; Wong et al., 2008). While some degree of controlled, conscious processing is necessary for older adults to maintain balance during walking (typically during more challenging conditions) (Boisgontier et al., 2013), excessive conscious movement processing could, however, jeopardize the control of gait through disrupting movement automaticity (Masters & Maxwell, 2008; Wulf, 2013). Mak and colleagues have illustrated that when older adults were instructed to consciously focus on their walking movements, poorer movement efficiency and gait stability accompanied by greater postural sway have been observed during a level-ground walking task (Mak et al., 2019, 2020).

In addition to hampering walking performance, conscious movement processing might induce older adults to adopt undesirable visual search behaviors, which in turn reduces their capacity to process and preview visual information on the surroundings or future pathway (Ellmers et al., 2020). Ellmers and colleagues observed that older individuals prioritized most of their gaze on the immediate area of the walkway over previewing to future stepping areas, as these visual behaviors were shown to be related to increased self-reported conscious movement processing (Ellmers et al., 2020). More importantly, lower stepping accuracy for future targets was also found. These findings inform us that older adults’ capacity to safely negotiate future environmental threats when walking in challenging conditions will likely be compromised by ineffective gaze and scanning behaviors (e.g., limited visual previewing) and thus increase the risk of falling (Young & Williams, 2015).

Considering the substantial impact of conscious movement processing on gait performance, investigating effective approaches that target mitigating the effect of conscious movement processing will be extremely beneficial for improving gait performance in older adults during rehabilitation. The use of “external focus” instructions has been recommended by psychomotor research in the literature to enhance motor performance. An external attentional focus was characterized as directing an individual’s attention to the movement effects on the environment (Wulf, 2013). When compared to directing the attention to body movements (an internal focus) or receiving no instructions, the use of an external focus has been consistently proven to promote more effective movement performance (Wulf, 2013) and such effectiveness has also been established in the older populations (Chen et al., 2023). For example, Chiviacowsky et al. (2010) conducted a study to investigate the influence of internal and external focus on the balance of older individuals. Participants were instructed to balance on a tilting platform using a stabilometer. The group instructed to focus externally (i.e., on keeping the markers on the platform horizontal) exhibited significantly better balance performance compared to the group instructed to focus internally (i.e., on maintaining the horizontal position of their feet). Using a similar theoretical concept, Huxhold et al. (2006) investigated how older adults control their standing posture and found that their postural performance improved when their attention was shifted away from maintaining posture to a simultaneous simple cognitive task (monitoring digits) which served a similar role of an external focus. The performance was better compared to when their attention was directed towards the execution of postural control (serving as an internal focus). Overall, the constrained action hypothesis posits that the disparities in performance observed between internal and external focus stem from variations in the underlying motor control mechanisms (Wulf et al., 2001). When individuals employ internally focused conscious movement processing, it disrupts the normal subconscious lower level processes and thus leads to inefficient movements (Clark, 2015; Masters & Maxwell, 2008; Wulf, 2013). Conversely, an external focus of attention promotes automatic control processes, enabling movements to be executed without interference from conscious processes.

When considering the association between attentional focus and conscious movement processing, previous work has indeed discovered that providing external focus instructions during walking tasks resulted in a significant reduction in real-time conscious processing of gait-related movements compared to an internal focus in older adults (Mak et al., 2021). It suggests that by focusing externally, the attention of the participants was effectively redirected away from their body movements with reduced dependence on conscious (verbal) control processes. Although the positive outcomes from previous research seem to imply that external focus manipulations likely enhance movement performance through the reduction in conscious movement processing in older adults, the effects of using external focus instructions as an interventional approach for improving gait remain uncertain. Therefore, our first aim was to investigate whether a single-session intervention (SSI) of external focus strategy could improve gait performance during an adaptive locomotion task in the older population. We specifically focused on gait variabilities (indicative of gait stability) since stride-to-stride fluctuations reveal crucial aspects of motor control, as well as the role of top-down cognitive control during walking (Hausdorff, 2005).

Our existing knowledge of the visual processes underlying the association between external focus and walking performance is still limited. As vision has been viewed as the most useful source of information in goal-directed movements (Carlton, 1981; Heath, 2005), Magill, (1998) argued that an external focus could direct visual attention towards ‘information-dense areas’ containing crucial environmental features that regulate how the body and limbs need to move to achieve the desired goal. Therefore, we also aimed to explore how the proposed external focus strategy could affect, if not optimize, visual attention in older adults. We hypothesized that the intervention would improve gait stability (represented by reduced gait variabilities and postural sway), while simultaneously altering visual search patterns that encourage feedforward planning for future walking areas (represented by spending more time gazing towards the destination rather than the immediate areas of the walkway). The findings from the present study could reveal a promising path for future large-scale research on psychomotor interventions for gait rehabilitation in older adults.

## Methods

### Participants

Previous research by Mak et al. (2020) reported an effect size of 0.17, which suggests a total sample size of 90 participants necessary to provide a 90% power (two-tailed alpha at 0.05) for this study. To account for an approximately 20% potential attrition rate, we recruited a total of 112 (56 per group) community-dwelling older adults (mean age = 71.3 ± 4.2) (Table 1). They were recruited from local community centers in Hong Kong by convenience sampling. All participants were aged 65 or above, and were able to walk independently indoors without walking aids. Participants were excluded if they had any history of untreated major neurological, vestibular or musculoskeletal disorder (e.g., Parkinson’s disease or stroke), acquired static visual acuity worse than 20/40 vision, or scored 23/30 or less on the Chinese version of the Mini-Mental State Examination (MMSE-C) (Chiu et al., 1994). The study was approved by the Institutional Review Board of the Hong Kong Polytechnic University (PolyU IRB) (reference number: HSEARS20210525002). Informed consent was obtained from all participants prior to any experimental procedures.

### Procedures

Participants were allocated randomly to one of two groups, an external focus group (EXT, *n* = 56) and a control group (CON, *n* = 56). The participants’ demographic information was acquired and baseline characteristics (i.e., balance ability, functional mobility, and falls efficacy) were assessed using a series of validated and reliable assessment tools. Balance ability was evaluated using the Berg Balance Scale (BBS) (Berg et al., 1989). BBS is a valid and reliable scale in which participants performed a total of 14 balancing tasks; a better balance ability is indicated by a higher score. Functional mobility was assessed by the Timed Up & Go (TUG) test, in which participants were asked to stand up from a chair and walk for 3 m, and then turn around and walk back to the chair and sit down (Shumway-Cook et al., 2000). All participants completed two trials of the TUG and the average time of these two trials was recorded. A completion time of more than 14 s indicates a high fall risk. Falls efficacy was assessed using the Chinese version of the Falls-Efficacy Scale International (FES-I (Ch) (Kwan et al., 2013); a 16-item scale that evaluates participants’ level of concern about falling when performing different daily living activities. A higher score represents higher concern of falling.

Before the SSI phase, all participants were invited to perform an obstacle circumvention walking task along an 8-m level-ground walkway for five consecutive trials as pre-intervention (T0). The general instruction for the walking task was to complete the trial at a natural walking pace. They had to get around an obstacle (diameter 1 m x height 2 m) located in the middle of the 8-m walkway (4 m away from the starting point) (Paquette & Vallis, 2010) (see Supplementary Fig. 1). Participants were instructed to keep their attention on the obstacle until a light cue indicated which direction (i.e., left or right, randomized) to circumvent the obstacle. The light cue was placed on the obstacle at the height of 1.4 m facing towards the starting point of the walkway so that participants could gaze at it at the start (see Supplementary Fig. 2). The light cue was activated when the participant reached 2 m from the starting point (i.e., approaching the obstacle). Participants were free to look wherever they thought was necessary to safely avoid the obstacle after the indication of the light cue. They were required to walk an additional 4 m after circumventing an obstacle (i.e., to continue to walk after circumventing the obstacle). The destination of the walkway was not visible to participants before they began circumventing the obstacle.

The SSI phase consisted of 20 consecutive training trials, with a rest interval of at least 30 seconds between trials. The design of the training trial was identical to T0, in which all participants had to circumvent the same obstacle during each trial on the 8-m level-ground walkway. The design of the light cue signal was also the same as T0. During each trial, the instruction for EXT was to first focus on a random series of digits ranging from 0 to 9 displayed on a monitor placed at an angle behind the obstacle, and then focus on another series of digits on another monitor placed at the end of the walkway (destination) after they get around the obstacle (Mak et al., 2020, 2021). Three identical 27” LED monitor were utilized; two positioned on the left and right side behind the obstacle (serving as intermediate destinations), and one positioned at the end of the walkway. The digits were the same on all three monitors; both right and left monitors were displaying the numbers during the trial to avoid guiding the participants to a specific side in addition to the light cue. The numbers shown on the monitor were approximately 5.5 in. × 8.5 in. and displayed upon gait onset. Each number was displayed for at least 2 s to allow participants to have sufficient time to read them (Mak et al., 2020, 2021). As a manipulation check, participants were asked a yes-or-no question immediately following the trial to verify whether a specific digit appeared on the monitor. This ensured that participants maintained focus on the monitor throughout the trial. On the other hand, the only instruction for CON was to walk to the end of this walkway at your natural pace.

After the completion of the SSI phase, all participants were invited to perform the obstacle circumvention walking task along the walkway for five consecutive trials as post-intervention (T1) on the same day, and another five trials as retention (T2) one week after. The design and instruction for T1 and T2 were identical to T0. The primary outcome of gait stability and secondary outcome of visual search behaviors were collected at T0, T1, and T2 to evaluate the interventional effect.

### Outcome measures

#### Primary outcome - gait stability

Gait kinematics were obtained by a three-dimensional motion capture system (Vicon; Oxford Metrics Ltd., Oxford, UK). Nineteen ball-shaped reflective markers were attached to participants at specific bony landmarks (Mak et al., 2020). Multiple infrared cameras tracked the location of each marker at a sampling rate of 100 Hz. A low-pass, third-order Butterworth filter operating at 20 Hz was used to filter the marker position data. Heel contact was characterized as the heel marker’s local vertical minimum. Toe off was characterized as a significant departure from the toe marker’s local vertical minimum. A stride was considered as heel-to-heel contact of the same foot. Spatial and temporal gait parameters such as step length, step width, stride length, stride time, swing time, stance time, double support time, and gait speed were computed using a customized script written in MATLAB (R2015b; MathWorks Inc., USA). A sternum marker was used to record movement of the sternum in the medial–lateral (M-L) direction. A virtual marker, created by taking the average of the right and left greater trochanters, was used to record the movement of the pelvis in the M-L direction. The ranges of M-L excursion of the sternum and pelvis were determined. To account for gait initiation and termination, all kinematic data of the first and last meters of the walkway were not included for calculation (Brach et al., 2005). The mean and standard deviation (SD) for all gait parameters were averaged bilaterally across the five trials for T0, T1, and T2. The SD of the corresponding gait parameters was used to represent variability measures that indicated gait stability.

#### Secondary outcome– visual search behaviors

All participants were also fitted with a wireless eye tracker (Dikablis Eye Tracking Glasses; Ergoneers GmbH, Egling, Germany) with a tracking frequency of 60 Hz to measure visual search behaviors. The eye tracker was calibrated using a 4-point calibration. Visual fixations referred to a gaze that focused on a single location for 100 ms or longer (Patla & Vickers, 1997). Fixation locations were classified as one of the four areas of interest (AOI): “obstacle” (any part on the obstacle), “ground” (the walkway area on the floor either next to or after the obstacle), “destination” (any area on the wall at the end of the walkway), and “random” (any other areas not covered by the three AOI [e.g. equipment, cameras etc.]). These AOI were subsequently used to compute the number of fixation and fixation duration. For the number of fixation, it refers to the number of times that the gaze was fixed on each AOI for 100ms or longer. For the fixation duration, the duration measured for each AOI was normalized to the total duration of the individual trial by presenting it as the percentage of time spent fixating each AOI. The number of fixation and fixation duration percentage on each AOI were averaged across the five trials for T0, T1, and T2.

### Statistical analysis

Statistical analysis was performed using SPSS Statistics version 28.0 (IBM Corp, Armonk, NY, USA). Significance level was set at *p* < 0.05. Any between-group differences in baseline characteristics were compared using multiple independent samples t-tests or chi-square tests. For visual search data, each area of interest (AOI) was treated as one dependent variable, resulting in a total of eight dependent variables for visual data: four AOIs for fixation duration percentage and four AOIs for the number of fixations. The independent variables were Time (T0, T1, and T2) and Group (EXT and CON). The immediate interventional and retention effects on the outcome measures (gait stability and visual search behaviors) were compared using 2 (Group) x 3 (Time) mixed-model ANOVA with post hoc Bonferroni corrections.

## Results

### Baseline characteristics

The participants’ baseline characteristics are shown in Table 1. The mean age was 71.31 ± 4.23 years and 71.4% were female. There were no significant differences in any of the baseline characteristics (i.e., age, cognitive ability, balance ability, functional mobility, and falls efficacy) between EXT and CON (all *p* > 0.05). The accuracy of the yes-no responses for the manipulation check in EXT was 93.6%. All participants successfully selected the correct direction as indicated by the light cue.

**Table 1:** Participants’ baseline characteristics

Variables	Mean (SD)			*p*-value
	Total (*n* = 112)	EXT(*n* = 56)	CON (*n* = 56)	
Age	71.3 (4.2)	71.7 (4.6)	71.0 (3.9)	0.362
Sex, female, n (%)	80 (71.4%)	42 (75.0%)	38 (67.9%)	0.403
With a history of falls, n (%)	71 (63.4%)	36 (64.3%)	35 (62.5%)	0.844
MMSE-C (range: 0–30)	29.1 (1.0)	29.1 (0.9)	29.2 (0.9)	0.451
BBS (range: 0–56)	51.9 (2.5)	51.9 (2.4)	51.9 (2.6)	0.969
TUG (seconds)	11.5 (2.3)	11.5 (2.3)	11.4 (2.3)	0.837
FES-I (Ch) (range: 16–64)	40.2 (12.6)	41.4 (13.1)	39.0 (12.1)	0.307

Note. MMSE-C = Mini-Mental State Examination (Chinese version); BBS = Berg Balance Scale; TUG = Timed Up and Go Test; FES-I (Ch) = Falls Efficacy Scale– International (Chinese version); EXT = external focus group; CON = control group

### Gait stability– body sway

**Fig. 1:**
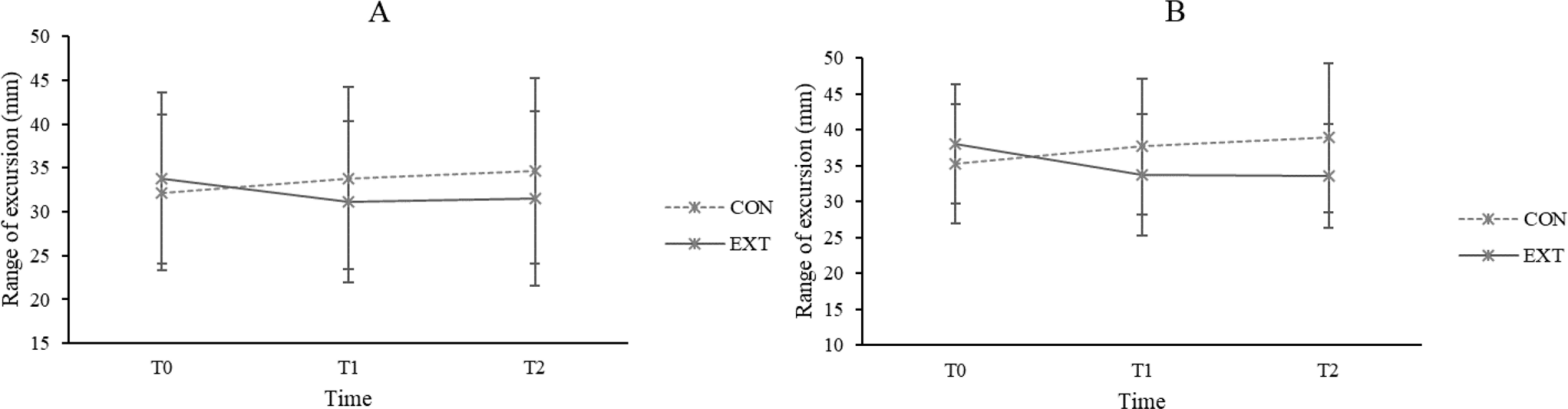
Range of medial–lateral excursion (in mm) of the sternum (**A**) and pelvis (**B**) regions at T0, T1, and T2 for external focus group (EXT) and control group (CON)

Results revealed significant interaction effects between Group and Time on the ranges of M-L excursion of the sternum and pelvis regions (sternum: F[1.805,198.510] = 15.553, *p* < 0.001, η_p_^2^ = 0.124; pelvis: F[1.798,197.762] = 43.984, *p* < 0.001, η_p_^2^ = 0.286). Given that there were no significant between-group differences for both variables at T0, post hoc analyses showed that EXT demonstrated significant reductions in the body sway of sternum and pelvis regions when comparing both T1 and T2 to T0 (all *p* < 0.05) (Fig. 1). On the other hand, CON showed significant increases in body sway at these regions when comparing T1 and T2 to T0 (all *p* < 0.05). There were no significant main effects of time and group on all variables (all *p* > 0.05).

### Gait stability– variability of gait parameters

**Fig. 2:**
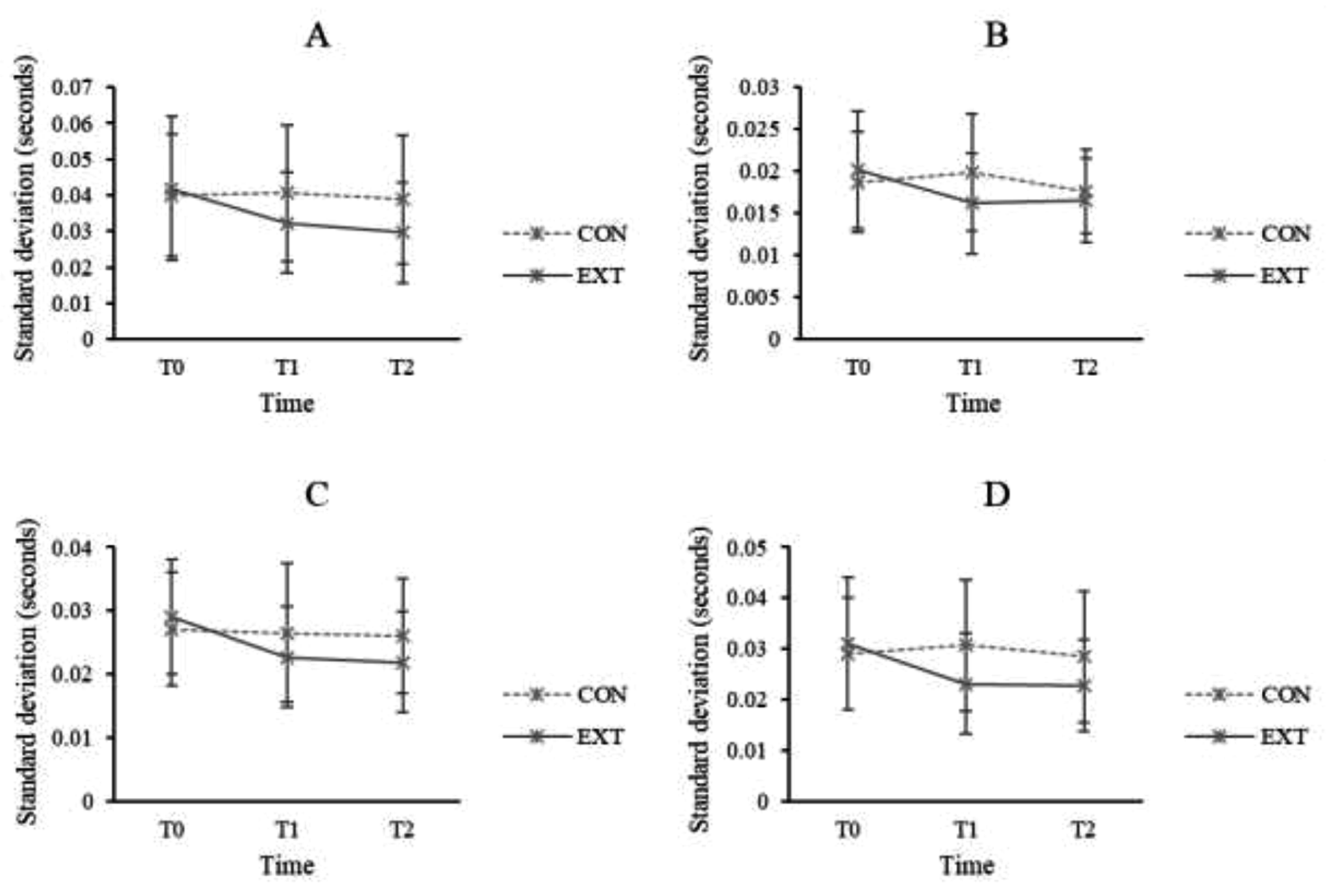
Variability of stride time (**A**), double support time (**B**), swing time (**C**), and stance time (**D**) (in seconds) at T0, T1, and T2 for external focus group (EXT) and control group (CON)

**Fig. 3:**
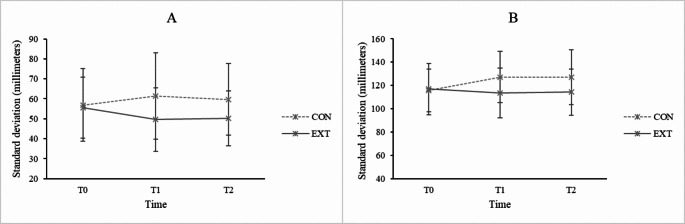
Variability of step length (**A**) and step width (**B**) (in millimeters) at T0, T1, and T2 for external focus group (EXT) and control group (CON)

Results revealed significant interaction effects between Group and Time on all variability of spatial and temporal gait parameters (stride length: F[2,220] = 10.322, *p* < 0.001 η_p_^2^ = 0.086; step length: F[1,110] = 7.330, *p* < 0.001, η_p_^2^ = 0.062; step width: F[2,220] = 10.698, *p* < 0.001, η_p_^2^ = 0.089; stride time: F[2,220] = 7.018, *p* = 0.001, η_p_^2^ = 0.060; swing time: F[2,220] = 10.868, *p* < 0.001, η_p_^2^ = 0.090; stance time: F[1.891,207.974] = 13.086, *p* < 0.001, η_p_^2^ = 0.106; double support time: F[2,220] = 10.552, *p* < 0.001, η_p_^2^ = 0.088; gait speed: F[2,220] = 4.312, *p* = 0.015, η_p_^2^ = 0.038). Given that there were no significant between-group differences for all variables at T0, post hoc analyses showed that EXT demonstrated a significant increase in gait speed and significant reductions in the variability of stride length, step length, stride time, swing time, stance time, and double support time when comparing both T1 and T2 to T0 (all *p* < 0.05) (Figs. 2 and 3). On the other hand, CON showed significant increases in variability of stride length and step width when comparing T1 to T0 as well as significant increases in variability of step width and gait speed when comparing T2 to T0 (all *p* < 0.05).

There were significant main effects of group on variability of stride length, step length, step width, stride time and stance time (all *p* < 0.05). Post hoc analyses showed that the variability in all these variables was greater in CON compared to EXT when averaged across all time points (all *p* < 0.05). There were significant main effects of time on variability of step width, stride time, swing time, stance time, double support time, and gait speed (all *p* < 0.05). Post hoc analyses showed that the variability of stride time, swing time, stance time, and double support time significantly decreased from T0 to both T1 and T2, while gait speed significantly increased from T0 to both T1 and T2 when averaged across all groups (all *p* < 0.05). The variability of step width significantly increased from T0 to T1 when averaged across all groups (*p* < 0.05).

### Visual search behaviors

**Fig. 4:**
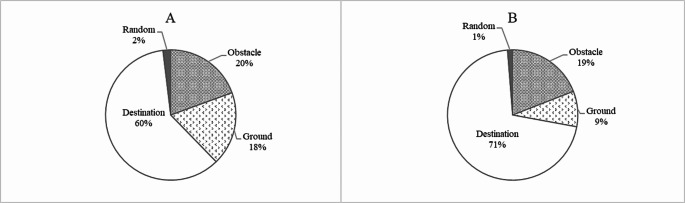
Percentage of fixation time spent on each of the identified areas of interest for external focus group (EXT) at T0 (**A**) and T1 (**B**)

Results revealed significant interaction effects between Group and Time on the number of fixation on ground and the fixation duration percentage on ground and destination (number of fixation on ground: F[2,220] = 16.202, *p* < 0.001, η_p_^2^ = 0.128; fixation duration percentage on ground: F[2,218] = 32.021, *p* < 0.001, η_p_^2^ = 0.225; fixation duration percentage on destination: F[1.810,197.326] = 24.244, *p* < 0.001, η_p_^2^ = 0.181). The significant differences in the fixation duration percentage on ground and destination at T0 were controlled as covariates for their interaction effects above. Post hoc analyses showed that EXT demonstrated significant reductions in the number of fixation on ground and fixation duration percentage on ground, accompanied by a significant increase in fixation duration percentage on destination when comparing both T1 and T2 to T0 (all *p* < 0.05) (Fig. 4). On the other hand, CON showed a significant increase in the number of fixation on ground when comparing T2 to T0, as well as a significant increase in fixation duration percentage on ground and a significant decrease in fixation duration percentage on destination when comparing both T1 and T2 to T0 (all *p* < 0.05) (Table 2).

There were no significant main effects of group on all variables (all *p* > 0.05). There was a significant main effect of time on the number of fixation on obstacle (*p* < 0.001). Post hoc analyses showed that the number of fixation on obstacle significantly decreased from T0 to T2 when averaged across all groups (*p* < 0.05).

**Table 2:** Fixation duration percentage on each of the identified areas of interest between EXT and CON under T0, T1, and T2

	Mean (SD)			Mean (SD)		
	EXT			CON		
	T0	T1	T2	T0	T1	T2
Obstacle (%)	19.09 (8.80)	18.89 (8.52)	17.39 (7.40)	18.82 (6.27)	19.74 (6.13)	19.79 (8.60)
Ground (%)	17.95 (14.73)	8.73 (11.14)	11.12 (11.15)	11.51 (10.51)	15.15 (13.54)	16.87 (13.05)
Destination (%)	59.24 (12.72)	70.11 (11.60)	66.92 (14.52)	66.00 (11.31)	61.79 (13.44)	58.71 (14.73)
Random (%)	1.88 (3.00)	1.25 (2.06)	1.42 (2.38)	2.89 (6.55)	2.73 (6.55)	2.06 (4.19)

Note. EXT = external focus group; CON = control group

## Discussion

The present study evaluated whether an external focus strategy could improve gait stability in an adaptive locomotion task in the older population. Our findings discovered that when compared to a control group, older individuals that received an external focus gait practice significantly improved stability in terms of reducing body sway and variability in spatial and temporal gait parameters; this effect was also observed at retention. Low variability of gait parameters is an index that can indicate consistency in walking movements and reflect the automatic process of rhythmic motor control related to gait safety with less cognitive effort (Dubost et al., 2006; Newell & Corcos, 1993). Matching with our prediction, practicing with the external focus manipulation seemed to successfully divert the attention away from participants’ body movements with more reliance on automatic control mechanisms and enhance movement fluency, as suggested by the constrained action hypothesis (Wulf, 2013). Such fluency has also been translated into better postural control, as indicated by a less degree of body sway when negotiating obstacles; an important factor to minimize fall risk by reducing the likelihood of hitting environmental constraints during circumvention.

We also aimed at exploring how the current psychomotor strategy could affect visual attention. Our findings indicate that gait improvements in EXT have been accompanied by a shift in visual search behaviors towards promoting feedforward planning for upcoming walking areas; a pattern that resembles how low-risk older adults (and young adults) exhibit during more complex walking conditions (Ellmers et al., 2020). Specifically, the reduced fixation duration on the ground in EXT aligns with the hypothesis that an external focus promotes movement automaticity, reducing the need for conscious processing of immediate stepping areas (Wulf, 2013). Conversely, the increased fixation duration on the destination suggests that older adults who practiced with the external focus manipulation tend to display more frequent proactive patterns of visual exploration by visually prioritizing distal areas of the walking path. It is interesting to note that fixation durations on the destination exceeded 50% of the trial duration—a seemingly high percentage given the walkway design—which may reflect anticipatory visual behavior initiated earlier in the walking path. As participants approached the side of the obstacle, lateral head/body movements likely enabled them to glimpse the destination before passing the obstacle. Building on these findings, we postulate that, in addition to promoting automaticity of movement, practicing with an external visual stimulus also improves gait by potentially fostering a more efficient planning of the walking path; prompting older adults to continually assess the upcoming environment during walking. This allowed walkers to survey the location of the next immediate and forthcoming steps, providing additional time to coordinate appropriate and consistent stepping responses (Yamada et al., 2012). Overall, the current observation reinforces the narrative that when walkers sample relevant environmental information in a feedforward manner through an external visual focus, it allows them to have sufficient time to adjust proactively for safe navigation around environmental hazards (Matthis & Fajen, 2014).

A previous systematic review has summarized that most of the existing research that investigated the effects of different attentional focus instructions on motor performance were observational studies (Chen et al., 2023), in contrast to the current study which involved a training component. In addition, only a few of them have focused on improving walking performance. For instance, Lövdén and colleagues (2008) examined the effect of a secondary cognitive task (serving a similar function of an external focus) on gait variability in older adults walking on a treadmill. Their findings indicated that gait variability was lower under the simple cognitive task compared to when older adults walked without any cognitive tasks. Interestingly, the simple cognitive task in their study involved the engagement of working memory (an n-back task) without visual processing demands, in contrast to the task used in this study. Although the mechanism of walking on a treadmill is different from level-ground walking, these findings collectively raise a question of whether an improvement in walking performance could be contributed by cognitive activity alone, visual inputs alone, or a combination of both. While this assumption requires further exploration, both processes nonetheless serve the function of experimentally withdrawing an internal attention from or limiting the opportunities for cognitive involvement in the movement control processes, which allows the motor system to self-organize and enhance movement automaticity (Huxhold et al., 2006).

As there is a scarcity of research in the literature that adopted external focus manipulation as a training strategy to improve balance or gait in the older population, the current work provides important insight into a causal link between external focus strategy and improved gait stability; an association presumably underpinned, at least in part, by changes in visual search patterns during adaptive locomotor tasks. The execution of motor tasks initiated by visual stimuli—such as navigating around a puddle—involves three fundamental stages of information processing: Stimulus Perception, Response Selection, and Response Execution (Gottsdanker & Shragg, 1985; Hommel, 1997; Koch et al., 2018). It is plausible that external focus facilitated participants’ ability to perceive environmental stimuli, such as obstacles, by enhancing their attention to relevant visual cues. This improved perception likely contributed to more effective selection of motor responses, thus optimizing decision-making during adaptive walking tasks. Consequently, enhanced perception and response selection may have resulted in more fluid and coordinated movements, thereby increasing gait stability through practicing with an external focus. These theoretical insights on perception, response selection, and motor execution highlight the potential of external focus interventions to improve gait performance and mitigate fall risk among older adults. The significant adjustments or improvements in visuomotor control also let us ponder the possibility that the current psychomotor strategy can also be applied to other cohorts exhibiting restricted visual search patterns which likely impair movement planning, such as those with movement difficulties, greater fear of falling, and/or a higher risk of falling (Ellmers et al., 2020).

For practical implications, physical therapists frequently use verbal communication during treatment sessions and practicing rehabilitation tasks. While most of this communication consist of feedback and/or instructions, a clear trend has been identified towards the adoption of internally focused information during rehabilitation (i.e., prompting patients to think explicitly about their body movements) (Durham et al., 2009; Johnson et al., 2013). Considering that bodily focus strategies might not be the most effective rehabilitative approach to impact walking performance in the geriatric population as discussed by Mak et al. (2020), clinicians can, instead, consider the implementation of an external focus approach (or similar psychomotor instructions that distract from body movements and promote visual previewing, e.g., the use of visual cues) as an adjunctive gait re-education intervention in rehabilitation settings in view of our current evidence.

A number of limitations should be considered. Although clear between-group differences were discovered in the relative absence of physical disparities, the current findings can yet to be extrapolated to older individuals with diminished physical conditions due to the relatively high functional ability of both groups. Second, while the current study adopted a single-session training paradigm to explore a novel psychomotor strategy that might hold promise for gait rehabilitation (Orrell et al., 2006), this short-term exposure and/or frequency of training might not be optimal to form a structured protocol for clinical practice. Nonetheless, we argue that even the current short-term treatment can result in significant improvements, a longer exposure of a structured intervention would likely result in greater benefits. Future study, especially a randomized controlled trial, is still warranted to determine the optimal interventional dosage and assess its long-term effects, including retention over a longer period (e.g., 6-month to 12-month). Third, our study did not include measurements of the precise timing of visual fixations in relation to specific gait events. Future research should aim to incorporate methods that allow for this level of detail to better understand the interaction between visual processing and gait dynamics. Another limitation is the controlled nature of the motor tasks used in training, which may not fully capture the adaptability required for diverse real-world scenarios. Future investigations could explore the implementation of varied motor tasks to enhance the ecological validity of the findings.

In conclusion, the current study represents the first attempt to provide concrete evidence about the potential benefits of an external focus strategy for gait rehabilitation. Results demonstrate positive behavioral improvements in gait stability (i.e., reduced body sway and variability in spatial and temporal gait parameters) accompanied by relatively desirable visual patterns that promote feedforward planning in the external focus group. From a clinical perspective, we advocate that gait practice with psychomotor manipulations that distract from body movements and/or promote visual previewing could potentially improve gait stability, especially under adaptive conditions. Further work might be necessary to establish optimal training dosage to enhance the design for a larger-scale psychomotor intervention for older adults.

## Electronic supplementary material

Below is the link to the electronic supplementary material.

Supplementary Material 1

Supplementary Material 2

Supplementary Material 3

## Data Availability

Data supporting this study’s findings are available at [https://osf.io/ckbjp/?view_only=6da49c2e899e41f1aa686f49a2e733fa].
