# Supplementary material for: External focus strategy improves visuomotor control of gait in older adults
Source: Psychol Res. 2025 Apr 23;89(3):95. doi: 10.1007/s00426-025-02122-3 (PMC12014841; doi:10.1007/s00426-025-02122-3)
Supplement: Supplementary file 1 — Supplementary Material 1 [file 426_2025_2122_MOESM1_ESM.docx]

**Supplementary Table 1.** General gait parameters (in mean) between EXT and CON under T0, T1, and T2.

|  | **Mean (*SD*)** | | | **Mean (*SD*)** | | |
| --- | --- | --- | --- | --- | --- | --- |
|  | EXT | | | CON | | |
|  | T0 | T1 | T2 | T0 | T1 | T2 |
| Stride time (s) | 1.128(0.136) | 1.098(0.116) | 1.100(0.1116) | 1.098(0.095) | 1.081(0.095) | 1.076(0.096) |
| Double-support time (s) | 0.179(0.040) | 0.173(0.033) | 0.172(0.032) | 0.172(0.026) | 0.169(0.028) | 0.163(0.026) |
| Stance time (s) | 0.742(0.104) | 0.722(0.086) | 0.721(0.084) | 0.720(0.069) | 0.708(0.070) | 0.701(0.071) |
| Swing time (s) | 0.385(0.037) | 0.376(0.033) | 0.378(0.034) | 0.376(0.032) | 0.370(0.032) | 0.374(0.031) |
| Stride length (mm) | 1031.173  (115.310) | 1076.815  (119.812) | 1071.048  (120.089) | 1047.144  (117.445) | 1050.380  (125.133) | 1061.863  (125.084) |
| Step length (mm) | 515.586  (57.654) | 538.408  (59.905) | 535.523  (60.043) | 523.571  (58.723) | 525.193  (62.570) | 530.929  (62.542) |
| Step width (mm) | 183.350  (26.128) | 177.257  (23.724) | 181.157  (27.241) | 186.469  (29.954) | 186.520  (29.069) | 190.694  (30.213) |

*Note.* EXT = external focus group; CON = control group.
