# Supplementary figures and images for: External focus strategy improves visuomotor control of gait in older adults
Source: Psychol Res. 2025 Apr 23;89(3):95. doi: 10.1007/s00426-025-02122-3 (PMC12014841; doi:10.1007/s00426-025-02122-3)

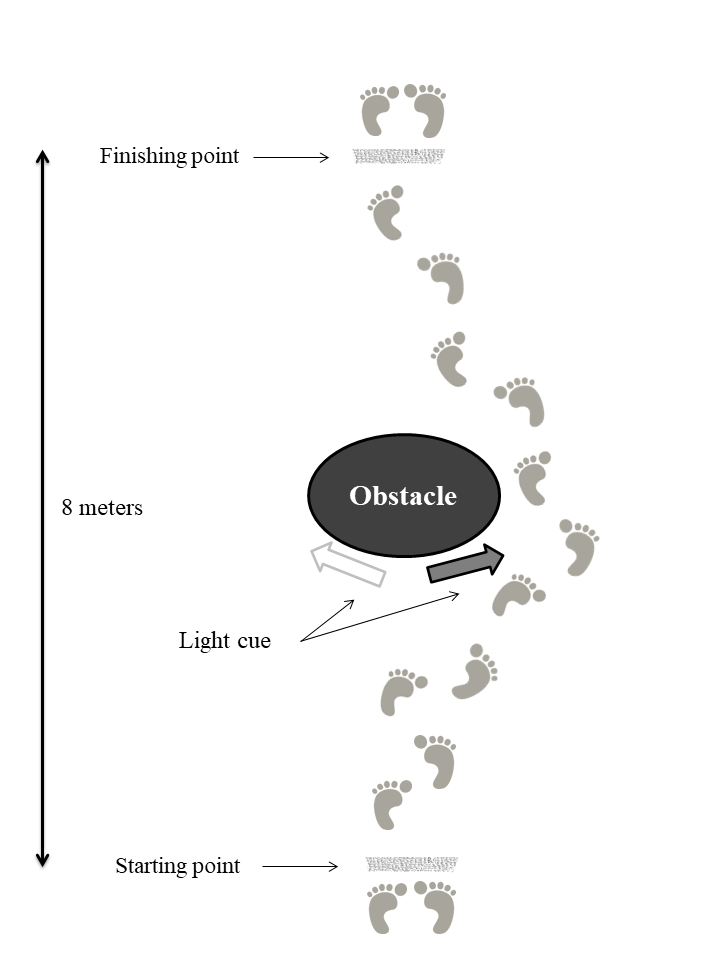

Supplement: Supplementary file 2 — Supplementary Material 2 [file 426_2025_2122_MOESM2_ESM.png]

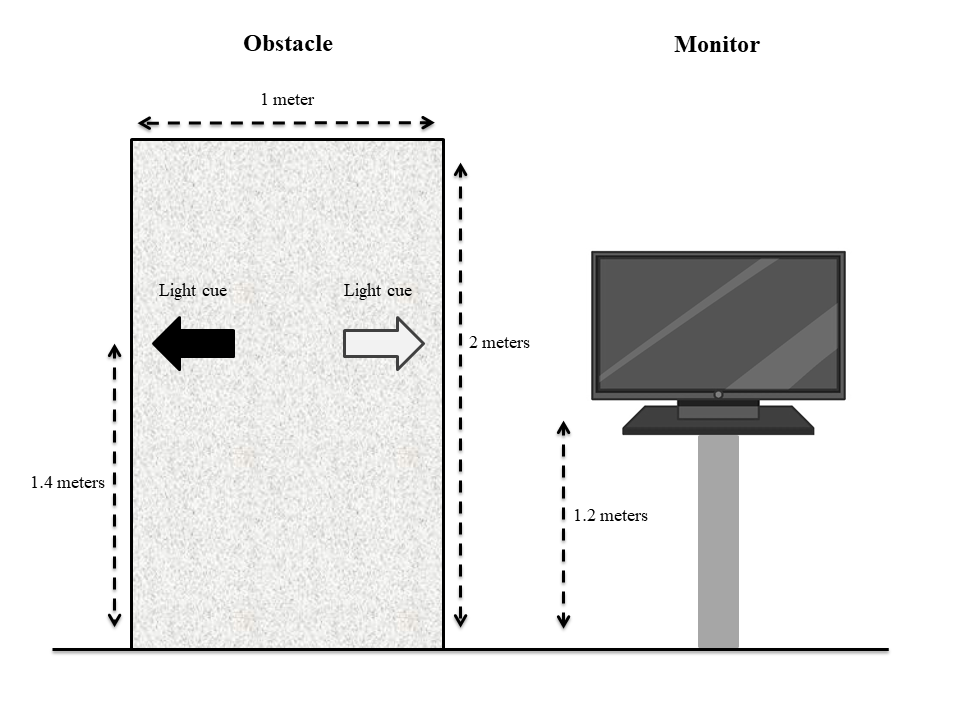

Supplement: Supplementary file 3 — Supplementary Material 3 [file 426_2025_2122_MOESM3_ESM.png]
